# Two-Dimensional V_2_O_5_ Inverse Opal: Fabrication and Electrochromic Application

**DOI:** 10.3390/ma15082904

**Published:** 2022-04-15

**Authors:** Hua Li, Zijuan Tang, Yuwei Liu, Jacques Robichaud, Jian Liang, Weihui Jiang, Yahia Djaoued

**Affiliations:** 1 Department of Materials Chemistry, School of Materials Science and Engineering, Jingdezhen Ceramic University, Jingdezhen 333403, China; h2jjsb@hotmail.com (H.L.); 1720021030@stu.jcu.edu.cn (Z.T.); 201002@jci.edu.cn (Y.L.); 2 Laboratoire de Recherche en Matériaux et Micro-Spectroscopies Raman et FTIR, Université de Moncton-Campus de Shippagan, Shippagan, NB E8S1P6, Canada; jacques.robichaud@umoncton.ca; 3 National Engineering Research Centre for Domestic & Building Ceramics, Jingdezhen Ceramic Institute, Jingdezhen 333001, China; liangjian@jci.edu.cn (J.L.); whj@jci.edu.cn (W.J.)

**Keywords:** vanadium oxide, two-dimensional inverse opal, ‘dynamic hard template’ strategy, electrochromic properties

## Abstract

The open-layered structure of Vanadium pentoxide (V_2_O_5_) has triggered significant interest in exploring its energy-related application as lithium (Li) intercalation cathode material. Various methods are extensively studied to improve the Li diffusion using thin films or nanoarchitecture. In this work, high-quality two-dimensional (2D) inverse opal α-V_2_O_5_ films were synthesized via a modified ‘dynamic hard template’ infiltration strategy using sacrificial polystyrene spheres (PS, a diameter of 530 nm) photonic crystal as a template. The new material exhibited an excellent porous array with featured structural colors in a large area. The electrochromic behavior was explored by combining bandgap and electrochemical characterization. On the one hand, the intercalation/deintercalation of Li^+^ played an important role in the bandgap (Eg), and thereafter on the visible range transmittance through changing the film’s stoichiometry and the valence of vanadium ions. On the other hand, the asymmetry of the lattice due to the disordered distribution of Li^+^ within the V_2_O_5_ interlayer and/or the formation of an irreversible phase explained the change in transmittance with voltage.

## 1. Introduction

Electrochromism has attracted much attention due to its commercial application in smart windows, displays and rearview mirrors [1]. Various known electrochromic materials include cathodic materials, which are colored in the reduced state and bleached in the oxidized state, anodic materials, which showed the opposite behaviour, and permanent color materials, which exhibited a different color between the oxidized and the reduced states [2,3].

Among them, vanadium pentoxide (V_2_O_5_), possessing an orthorhombic open-layered structure and therefore allowing small alkali metal ions like Li^+^ to be reversibly inserted, has triggered significant interest in the exploration of its energy-related-application as cathode material, such as electrochemical pseudocapacitors, electrochromic devices and electrooptic switches [1,2,3,4,5,6]. Through intercalation/deintercalation of Li^+^ ions within the interlayer sites, under increasing voltages [7], a series of phase transformations occurs in V_2_O_5_ bronzes from α, to ε, and δ phases. This phase change is accompanied by a change in the valence of vanadium ions from +5 to +3 and a volume change of up to 25% per V_2_O_5_ unit [8]. Importantly, if such a phase change relates to non-stoichiometry, which often exists in films, its electrochromic response would thus change greatly since oxides are strong electronic-correlation systems [9].

Despite its great prospect as lithium intercalation cathode material, practical application of V_2_O_5_ is hindered by limited rate performance and poor cycling stability due to its moderate electronic conductivity, intrinsically low ionic diffusion [10,11], and thereafter, irreversible phase transitions [12,13]. In this case, various methods are extensively explored, such as expanding interlayer distance by hydrated V_2_O_5_ [14] to improve ionic diffusion; using thin films [15] or nanoarchitecture [16,17,18,19,20], such as amorphous V_2_O_5_ with partial short-range order to reduce ionic diffusion distance and to increase reaction sites [21]. Recently, three-dimensional macroporous [22,23,24] or microporous architectures [25], especially inverse opal (IO) as a highly ordered porous array structure with V_2_O_5_ frameworks, have shown great potential due to their large active surface areas within a continuously interconnected electrode structure and excellent strain-accommodation of porosity. The strain arises from the volume change during Li^+^ intercalation/deintercalation. Inverse opal is a highly ordered microporous structural material with thin walls and a high surface area to volume ratio [26]. From the electrochromic point of view, the nanosized wall provides a short diffusion distance, rendering a high switching speed, while a large area gives continuous pore space for good electrolyte penetration, endowing sufficient electroactive sites for intercalation/deintercalation of Li^+^, and thereby large color modulation [4]. Although inverse opal (IO) is advantageous over traditional flat films, the reports on synthesis are limited [1,4,22,23]. Almost all of these reports utilize synthesis through the electrochemical route (electrodeposition and electrophoretic deposition). Under this route, to strengthen the adhesion between polystyrene spheres (PS) opal template and the ITO glass substrate and avoid the PS film from peeling off the ITO surface due to the surface tension of the electrolyte solution (vanadium ion precursor’s aqueous or ethanol–aqueous mixture solution), the preheat treatment of the PS opal film is required [1].

We have previously developed an efficient method—a “dynamic hard template” strategy—for fabricating large-area, crack-free, two-dimensional (2D) inverse opal (IO) films, including WO_3_ [27], TiO_2_ [28], VO_2_ [29], TiO_2_/WO_3_ multilayer [30] and so on, and have further explored their chromic response. In this strategy, the oxide precursors/PS opal composite film was primarily fabricated in a lotus-leaf style floating on water following the formation of PS opal film at the water surface as a dynamic hard template [28]. Afterward, this composite film was sunk onto ITO substrates by sucking out water. An oxide IO film was finally obtained simply by calcination, which served two purposes: converting the oxide precursors into the desired oxide; inversion of the opal film by removing the PS spheres template. In the case of V_2_O_5_, since the infiltration of oxide precursors into PS opal template was conducted before it was deposited on indium-doped tin oxide-coated glass substrates (ITO substrates), the effect of surface tension from V_2_O_5_ precursor solution on the PS film adherence to the ITO template was avoided. Therefore, large-area, crack-free, two-dimensional (2D) oxides inverse opal films could be easily synthesized.

In the present study, we synthesized large-area crack-free 2D V_2_O_5_ inverse opal primarily by an improved dynamic hard template strategy and explored the relationship between electrochromism and structure by cyclic voltammetry (CV).

## 2. Experimental Materials and Methods

### 2.1. Materials

The non-cross-linked monodispersed carboxyl polystyrene particles aqueous suspensions (PS particles, nominal size 530 nm in diameter, 5.0% *w*/*v*) were purchased from Spherotech Inc., Lake Forest, IL, USA. Before use, they were diluted to 0.5% *w*/*v* with volume ratio (water: ethanol) of 1:1. ITO slides were ultrasonically treated for 15 min in warm water, acetone, ethanol and deionized water, respectively. NH_4_VO_3_, analytically pure tetrahydrofuran (THF), sodium dodecylsulfate (SDS) and Millipore water were purchased from Sinoreagent Co. Ltd. and used as received without further purification. The aqueous solutions were prepared with Millipore water (resistance = 18.2 MΩ cm^−1^). The glass slides were immersed in a piranha solution (30% H_2_O_2_: concentrated H_2_SO_4_ = 3:7, *v*/*v*) at 80 °C for 60 min then washed with Millipore water.

### 2.2. Fabrication of PS Monolayer Inverse Opal

Initially, a clean, functionalized glass slide was placed on the bottom of a petri dish. Then, Millipore water was added to just submerge the glass slide. Afterward, diluted PS suspension was added dropwise onto the glass slide to get a self-assembled monolayer of PS spheres on the water surface. Then, a few drops of 2 wt.% SDS aqueous solution was added into the water to closely pack the PS monolayer, resulting in a 2D PS opal template floating over the solution.

### 2.3. Fabrication of 2D-V_2_O_5_ IO Structure: Improved “Dynamic-Hard-Template” Infiltration Strategy

The V_2_O_5_ inverse opal films were obtained by an improved dynamic-hard template infiltration strategy developed in our previous work [27,28,29], using PS spheres of nominal diameters of 530 nm.

Once the PS opal was formed floating over the solution, most of the volume of water was removed, and 4 mL of water was left in the petri dish, while PS opal was still floating onto the water surface. Then, the PS opal was heated to 50–60 °C in a petri dish. Afterward, the NH_4_VO_3_ aqueous solution (0.3 g NH_4_VO_3_/20 mL water) was injected into the water just under the 2D PS opal film. After waiting several minutes for the system to stabilize, a 2D NH_4_VO_3_/PS opal composite film (named ‘VP opal composite’) was sunk onto ITO substrates by sliding the substrates into the water underneath the composite film and then sucking out the water. V_2_O_5_ 2D IO (named ‘V_2_O_5_ IO’) is obtained by annealing the NH_4_VO_3_/PS opal composite film at 310 °C for 1 h.

### 2.4. Characterization

Optical transmittance of the V_2_O_5_ thin films was recorded in the 300 to 1100 nm range using a Biochrom Ultrospec 2000 UV-vis NIR (Cambridge, UK) spectrophotometer (Cambridge, UK). The morphology of the films was characterised using a Hitachi S-4800 FE-SEM microscope (Ibaraki-ken, Japan). For phase analysis, Raman Spectra were recorded with a Jobin-Yvon LabRAM HR microanalytical spectrometer (Villeneuve d’Asq, France) equipped with a motorized XY stage and autofocus. The spectra were generated with ~0.45 mW, 633 nm He-Ne laser excitation at the sample surface. Filters were used to vary the laser power needed since V_2_O_5_ is sensitive to laser heating.

Cyclic voltammetry (CV) measurements were performed with a Gamry 3000 Potentiostat/Galvanostat/ZRA (Warminster, PA, USA) electrochemical workstation (Warminster, PA, USA) using a three-electrode cell in the voltage range from −2 to 2 V with 20 mV/s velocity. The V_2_O_5_ thin films deposited on the ITO substrate were used as working electrodes while a platinum grid served as a counter electrode, and a commercial Ag/AgCl 1 M KCl electrode served as a reference. A 1 mol/L LiClO_4_/propylene carbonate solution was used as an electrolyte.

## 3. Results and Discussion

Unlike our previously reported dynamic-hard-template infiltration strategy, conducted at room temperature [27], the present synthesis of V_2_O_5_ IO films was performed at a temperature of 50–60 °C [29]. The infiltration depends on the capillarity, which was affected by both the precursor (NH_4_VO_3_) aqueous solution and the interstitial space between the PS spheres. Once the water within the interstices among PS spheres evaporates, the precursor remains, forming a NH_4_VO_3_/PS opal composite. Therefore, the concentration of NH_4_VO_3_ aqueous solution plays a key role in forming a high-quality V_2_O_5_ IO film framework. Unfortunately, the extraordinarily low solubility of NH_4_VO_3_ at room temperature (0.48 g/100 mL, 20 °C) hinders the availability of a final V_2_O_5_ IO. In contrast, the solubility at 40 °C of NH_4_VO_3_ (1.32 g/100 mL at 40 °C) was high enough to get a proper thickness of the IO framework. Apart from it, considering that a glass transition and deformation of the PS template occurs at around 80–100 °C, the present synthesis was conducted at 50–60 °C.

**Figure 1:**
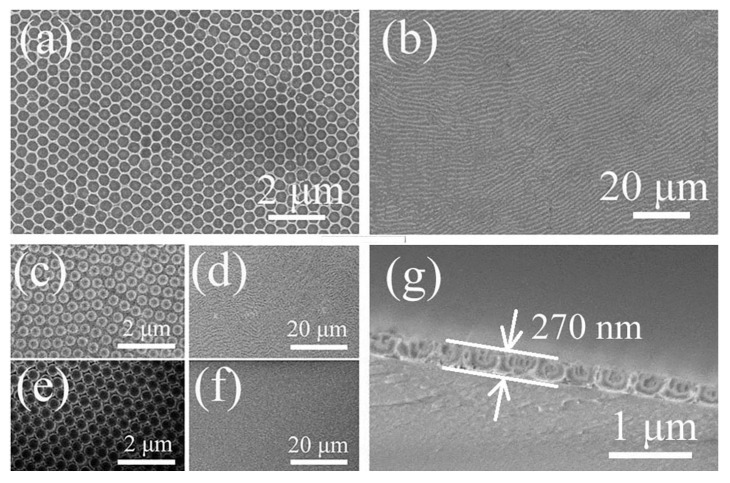
SEM images of (**a**,**b**) 2D V_2_O_5_ IO (**a**,**b**); VP opal composite (**c**,**d**); 2D PS opal template (**e**,**f**); and cross-sectional images of 2D V_2_O_5_ IO (**g**). Scale bars in (**a**,**c**,**e**) are 2 μm, 20 μm in (**b**,**d**,**f**) and 1 μm in (**g**).

Figure 1a–f show the SEM images of the large-area, crack-free (Figure 1b,d,f), highly ordered. Uniform opal structures (Figure 1a,c,e) obtained from 530 nm PS spheres at each fabrication step from PS opal (Figure 1e,f) to NH_4_VO_3_/PS (VP) opal composite (Figure 1c,d), to V_2_O_5_ IO (VIO) (Figure 1a,b). The opal periods of the samples were estimated from the SEM images using the Nanomeasurer software. The results were 443 nm, 451 nm, and 428 nm for sample PS opal, VP composite film and V_2_O_5_ IO, respectively. PS spheres were smaller than the nominal size of 530 nm, similar to our earlier observation [31]. The resultant VIO showed a shrinkage around 5% after calcination of VP opal composite film, partly due to mass loss and density increase during the transformation of the sample from 2D VP opal composite to VIO. Due to the high concentration of NH_4_VO_3_ precursor solution used during infiltration, NH_4_VO_3_ could be trapped within the interstitial of PS spheres. After the removal of the PS template by calcination, the framework was perfectly preserved, and a homogeneous porous array film with 270 nm thickness was obtained, as shown by the cross-sectional image of VIO (Figure 1g).

**Figure 2:**
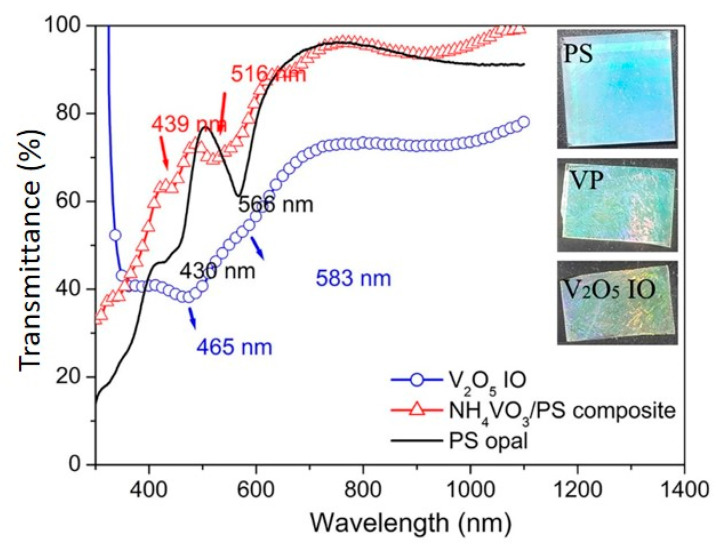
UV-vis-NIR transmittance spectra of PS opal, VP opal composite (red triangles), and V_2_O_5_ IO (blue circle). In the inset, optical images for PS opal (**top**, 25 mm × 25 mm), VP opal composite film (**middle**. 20 mm × 25 mm), and V_2_O_5_ IO (**bottom**, 15 mm × 25 mm) supported on ITO.

UV-vis NIR transmittance spectra of PS opal, NH_4_VO_3_/PS opal composite, and V_2_O_5_ IO monolayer films are shown in Figure 2. Similar to the observation of SEM, all samples showed large-area highly ordered array structures, noticeable from the reflection peaks. Reflection maxima appeared in the 430–470 nm range for the PS opal, NH_4_VO_3_/PS opal composite, and V_2_O_5_ IO monolayer films, rendering a blue color. The other reflection maxima for the PS opal and NH_4_VO_3_/PS opal composite occurred in the 510–560 nm range, corresponding to green color, while the next reflection maximum for the V_2_O_5_ IO monolayer film showed a slight red shift to 583 nm, which was within the light orange region (580–650 nm). Apart from it, the reflection showed a slight blue shift from PS opal to VP opal composite and then a red shift from VP opal composite to VIO monolayer films, which indicated that the opal structure period of the films increased and decreased, respectively. Such results agreed with the SEM observation (Figure 1), in which the opal structure periods were 443 nm for the PS opal, 451 nm for the VP opal composite, and 428 nm for V_2_O_5_ IO. The composite color from these two reflection regions was blue-green, which matched well with the optical photos (Figure 2, inset), demonstrating their structural blue-green color. After calcination, V_2_O_5_ IO showed blue-green-golden color, which indicated a combination of structural color and the original V_2_O_5_ orange-yellow color.

**Figure 3:**
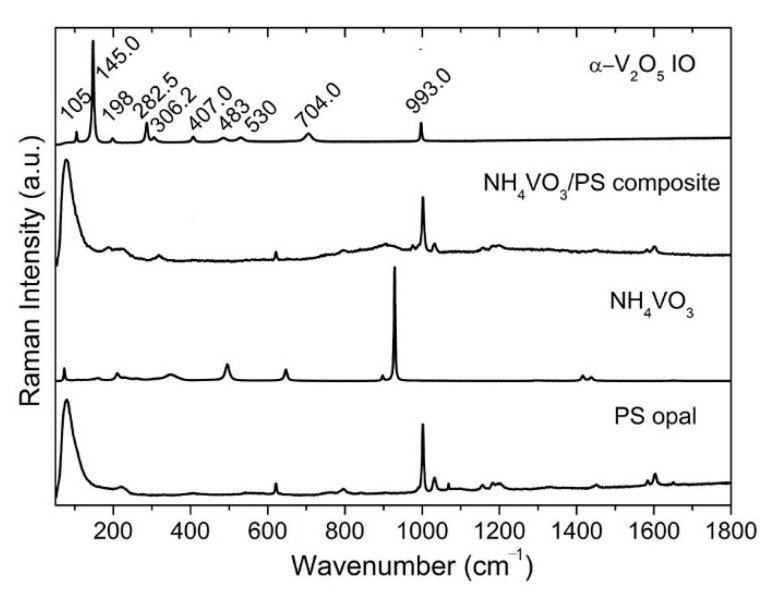
Raman spectra of PS opal, NH_4_VO_3_ powder, 2D NH_4_VO_3_/PS opal composite, and V_2_O_5_ IO.

As discussed above, UV-vis results show that a 2D V_2_O_5_ inverse opal was successfully obtained due to the important contribution of typical structural color from opal or inverse opal, based on two valleys around 430–470 nm and 510–560 nm in UV-vis-NIR patterns for all films (Figure 2). Moreover, SEM observations (Figure 1a,b) supported by a Raman spectrum (Figure 3) showed a highly ordered array of pores with a large-area V_2_O_5_ framework.

Raman spectroscopy is often used to analyze the crystallinity of inorganic materials [32,33,34]. Figure 3 shows the Raman spectra of the PS opal, the NH_4_VO_3_ powder, the 2D NH_4_VO_3_/PS opal composite and V_2_O_5_ IO. The characteristic Raman modes of α-V_2_O_5_ emerges at ~105, 145, 198, 282, 306, 407, 483, 530, 704, 993 cm^−1^. The low-frequency modes at ~105, 145 and 198 cm^−1^ corresponded to the relative motions of V_2_O_5_ layers (external modes) in V_2_O_5_ [33]. The two peaks at 145 and 198 cm^−1^ were strongly associated with the layered structure, demonstrating its long-range structural order [33]. The intermediate frequency peaks at ~282, 306, 407, 483, 530, 704 cm^−1^ related to the bending and stretching vibrations (internal modes) of vanadium–oxide bond in V_2_O_5_ [34]. The highest frequency peak at ~993 cm^−1^ corresponded to the stretching mode of the terminal oxygen (vanadyl oxygen, V = Oν). Similar to what was reported in the literature [33,34], the absence of a mode at 840 cm^−1^, which is Raman active in defective V_2_O_5_, confirmed good crystallinity of the V_2_O_5_ phase within the film.

**Figure 4:**
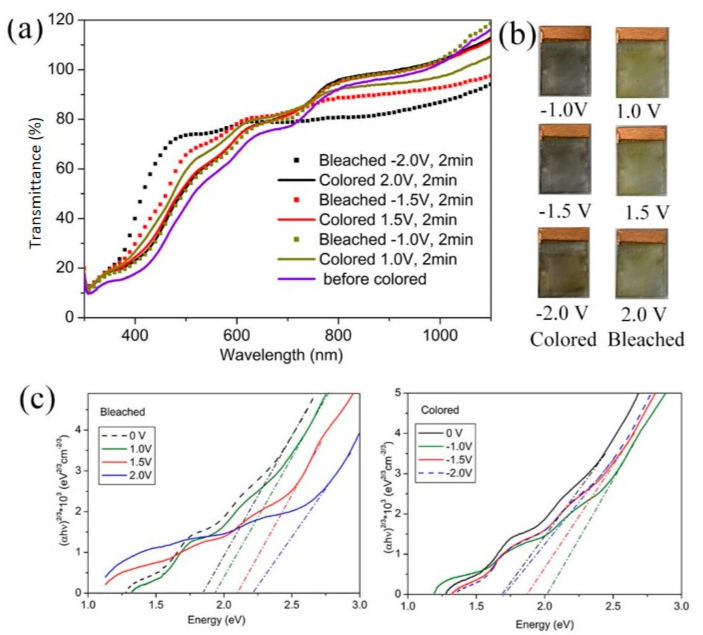
(**a**) Transmittance spectra; (**b**) optical photos in colored and bleached states; and (**c**), Tauc plots of V_2_O_5_ IO in the as-prepared state and under alternating applied potentials.

The electrochromic performance was further investigated. Figure 4a shows the optical transmittance of the V_2_O_5_ IO film in its colored and bleached states for applied coloration/bleaching potentials of ±1.0, ±1.5, 0 and ±2.0 V. As it is for most V_2_O_5_ films [3,4], the optical responses in bleached states were different from 0 V, i.e., the optical transmittance for the bleached state was not the same as at 0 V. In all of the colored states, the film appeared grey to black with a little red for −2.0 V, while in the bleached state, all films showed yellow color, similar to the intrinsic color of V_2_O_5_ powder (Figure 4b). It can also be noted that the electrochromic results in Figure 4 showed that, in the colored state, as the coloration deepened (−1.0 V, −1.5 V to −2.0 V), the transmittance decreased in the visible region and increased in the NIR region while, in a bleached state, as the bleaching voltage increased, the transmittance increased in the visible region and decreased in the NIR region.

Optical density (ΔOD) was used to study the optical modulation with light wavelength:

(1) $$\Delta OD=\log\left(T_{b}/T_{c}\right)$$

where T_b_ and T_c_ refer to the transmittance of the bleached and colored sample, respectively [35]. As seen in Table 1 and Figure S1 (see Supplementary Materials), the largest optical modulation for all voltages applied occurs at around 400–500 nm wavelength: −8% at 495 nm for ±1.0 V; 12.1% at 487 nm for ±1.5 V; 31% at 442 nm for ±2.0 V. In contrast, the optical modulations in the NIR region (1100 nm) were found to be 13.4% for ±1.0 V, −14.3% for ±1.5 V, 18.6% for ±2.0 V.

Generally, the conduction band in V_2_O_5_ is formed by the vanadium 3d bands and the valence band by the 2p bands of oxygen, which is a direct forbidden transition. The optical absorption coefficient α of the films was calculated using the equation:

(2) $$\alpha t=In\;\left(\frac{1}{T}\right)$$

where T is the transmittance and *t* is the thickness of the film.

Figure 4c shows the plots of ${\left(\alpha h\nu \right)}^{2/3}$ vs. hν. The optical bandgaps (Eg), which were evaluated by extrapolating the linear portions of the plots to zero, are provided in Table 2.

The film at 0 V showed Eg around 1.68 eV, far lower than stoichiometric V_2_O_5_, indicating that it could be non-stoichiometric [34], which was possibly due to the reduction of NH_3_ from the decomposition of NH_4_VO_3_ [36]. Combining a strong absorption in red light (from Eg absorption) and a strong reflection in blue-green from the structural array, the transmittance of the film at 0 V in visible light was fairly low, even much lower than all other films (Figure 4a). As the coloring voltage increased from −1.0 V to −2.0 V, which was a reductive process, the bandgap decreased. Such decreases were a reflection of both lithium ions intercalation and valence change of vanadium ions from +5 to +3: the decrease in valence increases the metallicity of vanadium oxide [37,38,39] and increases oxygen vacancies and/or the non-stoichiometry in the film [7,8] while the intercalation of lithium ions increased the interlayer distance, and therefore, increased the ions’ transportation. Similar to the literature [4], at the applied reductive voltage (−1.0 V, −1.5 V and −2.0 V), the film became black. We observed that the film colored at −1.5 V showed a transmittance similar to the film colored at −2.0 V in the whole light region, indicating that at both voltages, the films may have had a similar structure/distribution of Li^+^ among the interlayer of V_2_O_5_ and nearly the same amount of intercalated Li [8]. In the NIR region, one should see that the transmittance of the film colored at −1.0 V showed the lowest transmittance among all colored films. It is well-known that IR absorption is related to lattice vibration: high symmetry leads to low absorption of IR [40]. It is generally accepted that Li_x_V_2_O_5_ suffers from several metastable phase changes at room temperature as lithium content varies: for x < 0.1, a slightly distorted α-phase; for 0.35 < x < 0.8, ε-phase; and for 0.88 < x < 1.0, δ-phase. As the intercalation of Li^+^ goes on, a progressive puckering of the layers along the a–b planes occurs, leading to an enlarged interlayer spacing and a concomitant decrease in the a-parameter of the unit cell. Such distribution of Li^+^ within the interlayer of V_2_O_5_ results in disordered to ordered distribution since the sites available for Li^+^ intercalation are decreased as lithium content increases. Therefore, it could be deduced that at −1.0 V, a distribution of Li ions is in a disordered state, increasing the lattice’s asymmetry. As a result, under −1.0 V, the film showed the lowest absorption in IR.

As for the bleached states, which is an oxidation process, the bandgap in the visible region increased as the bleaching voltage was applied: considering that the film at 0 V could be non-stoichiometric V_2_O_5_, as the bleaching voltage increased, the stoichiometry was improved. This change increased the Eg and thus increased the transmittance in the visible region. However, the transmittance in the NIR region showed the opposite behavior.

**Figure 5:**
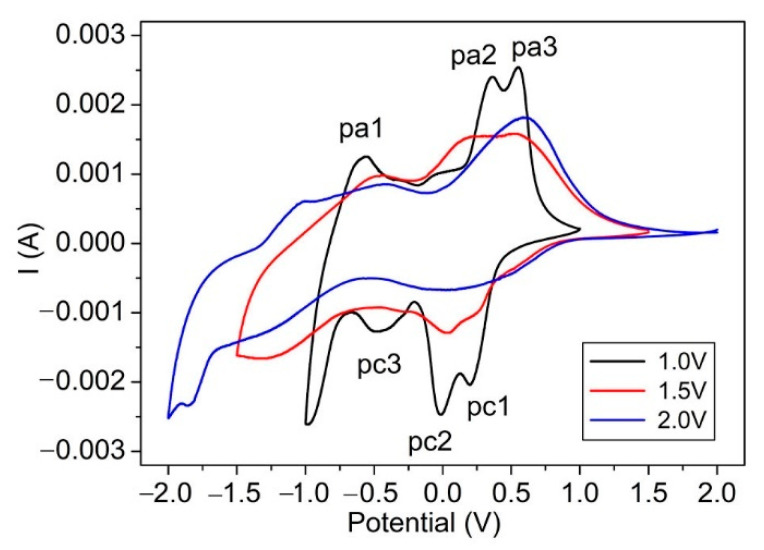
Room temperature voltammograms of V_2_O_5_ IO film at various potential windows in 1 mol/L LiClO_4_/propylene carbonate solution at a sweep rate of 20 mV/s.

**Figure 6:**
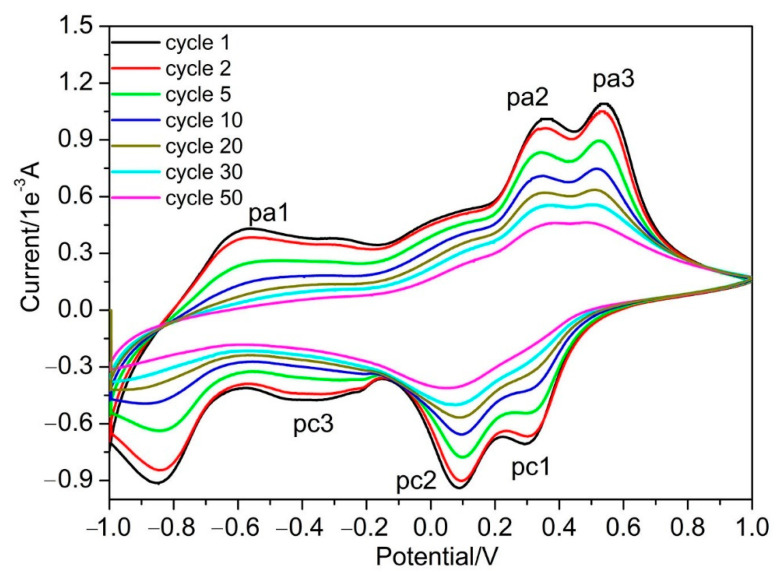
Room temperature long-term cycling voltammograms of V_2_O_5_ IO film at a potential window of ±1.0 V in 1 mol/L LiClO_4_/propylene carbonate solution at a sweep rate of 20 mV/s.

In order to understand this abnormality, room temperature voltammograms at sweep rates of 20 mV/s of V_2_O_5_ IO film were conducted at various potential windows (±1.0, ±1.5 and ±2.0), to understand the intercalation/deintercalation process of Li^+^ (Figure 5). The data were obtained at the fifth cycle, which shows a nearly constant cycle status. We found that the peaks in each cycle were nearly stable, except for the cycles above the 20th, after which the peaks tended to merge, as shown in Figure 6.

The CV of V_2_O_5_ IO film showed three cathodic reduction peaks (pc1, pc2, pc3) attributed to lithium intercalation and three anodic peaks (pa1, pa2, pa3), which corresponded to lithium extraction. Different peaks are ascribed to the formation of different crystalline phases of Li_x_V_2_O_5_ or successive redox/oxidic reactions [3,41]. The film under a potential window of ±1.0 V showed better-defined peaks, and there was no significant difference in the intercalation and deintercalation capacities or cathodic and corresponding anodic current, indicating its good reversibility. The overall shape of the CVs for the film at a potential window of ±1.5 V had some distortion in contrast to that under a potential window of ±1.0 V, and the three cathodic and anodic peaks broadened and merged together to form two peaks. This change became more obvious for the film at a potential window of ±2.0 V, where a vast asymmetry was observed, indicating its irreversibility as well as a deterioration of the film. Such irreversibility could be attributed to the formation of an irreversible rock-salt phase of ω-Li_3_V_2_O_5_ when cycled around 1.9 V [22] or a partial dissolution and/or a film delamination [3]. Due to the structure change and deterioration of the film—which increased with the bleaching voltage—the NIR absorption increased accordingly, which was consistent with the observation in Figure 4.

Based on CV results from various potential windows, further long-term cycling of the obtained V_2_O_5_ IO film was conducted with a potential window of ±1.0 V at a sweep rate of 20 mV/s (Figure 6). The overall shape of the CVs had high similarities, while the capacity decreased with the number of cycles. After the 30th cycle, the cathodic peaks of pc1 and pc2 merged, broadened and greatly decreased. The same phenomenon happened for anodic peaks pa2 and pa3—the pa1 and pc3 flattened, indicating that the capacity greatly decreased. Although the peaks decreased, the site for these peaks did not move and the symmetry of the CV curves was well-retained, indicating that the Li^+^ intercalation/deintercalation proceeded with the same V_2_O_5_ structure from the 1st to the 20th cycle. We found that the film partially peeled off the ITO substrates after the 30th cycle, which could be the reason for the decreased capacity of the film.

## 4. Conclusions

High-quality two-dimensional (2D) inverse opal (IO) α-V_2_O_5_ films were synthesized via a modified dynamic hard template infiltration strategy using sacrificial polystyrene (PS) spheres photonic crystal as a template (diameter of 530 nm). Due to the benefits of the dynamic hard-template infiltration strategy, IO films with an area as large as 15 × 25 mm^2^ were obtained. The obtained new material exhibited an excellent porous array with a featured structural blue-green color. Considering the intrinsic orange-yellow color of α-V_2_O_5_, the final α-V_2_O_5_ IO showed a blue-green-golden composite color.

The electrochromic properties of the IO films were analyzed by combining Eg and electrochemical properties. Eg decreased with coloring voltage and increased with bleaching voltage, while at 0 V, the EG was lower than that of the film at colored and bleached states. The low Eg of the film at 0 V was attributed to possible non-stoichiometry. As the coloring voltage was increased, the transmittance of the α-V_2_O_5_ IO in the visible region reduced due to the decrease in Eg, which was due to the decrease in the valence of Vanadium ions, increase in oxygen vacancies and increased non-stoichiometry of the films as well. All of the films colored from −1.0 V to −2.0 V showed similar gray to black color. As for the transmittance, in the NIR region for colored states, transmittance of the α-V_2_O_5_ IOs increased, which could be explained through the improved ordering in the distribution of Li^+^ within the interlayer of V_2_O_5_ and the symmetry of the lattice, which was experienced from disordered to ordered as the coloring voltage increased.

As the bleaching voltage increased, the transmittance in the visible region of α-V_2_O_5_ IO increased due to the improved stoichiometry of the film under such an oxidation process, which increased the Eg accordingly. In contrast, the transmittance in the NIR region for bleached states was found to decrease with the bleaching voltage, which could be attributed to the irreversible deterioration of the lattice structure in the film under high voltage, such as 2.0 V.

A long-term cycling CV at a potential window of ±1.0 V proved that the obtained film capacity decreases with cycling, partially due to the film peeling off from the ITO substrates.

## Figures and Tables

**Table 1:** Electrochromic optical modulation at different voltages.

Voltage (V)	Biggest Contrast		$\Delta T_{450}\;(\%){\;}^{a}$	$\Delta T_{1100}\;(\%){\;}^{b}$
	Wavelength (nm)	ΔT (%)		
± **1.0**	495	−8	−6.9	13.4
± **1.5**	487	12.1	8.9	−14.3
± **2.0**	442.5	31	30.8	−18.6

^a^ ${\Delta T}_{450}$ (%): optical contrast in light wavelength of 450 nm; ^b^ ${\Delta T}_{1100}$ (%): optical contrast in light wavelength of 1100 nm.

**Table 2:** Changes in the optical Eg with applied potential.

Applied Potential (V)	Eg (eV)	Applied Potential (V)	Eg (eV)
**0**	1.68	0	1.68
**−1.0**	2.0	1.0	1.93
**−1.5**	1.86	1.5	2.15
**−2.0**	1.70	2.0	2.21

## Data Availability

Not applicable.

## Supplementary Materials

The following supporting information can be downloaded at: https://www.mdpi.com/article/10.3390/ma15082904/s1, Figure S1: Optical density of V_2_O_5_ IO film under variant voltage applied.

## References

[1] Li L., Steiner U., Mahajan S. (2010). Improved electrochromic performance in inverse opal vanadium oxide film. J. Mater. Chem..

[2] Granqvist C.G. (1995). Handbook of Inorganic Electrochromic Devices.

[3] Salek G., Bellanger B., Gaudon I.M., Rougier A. (2016). Polyol Synthesis of Ti-V_2_O_5_ Nanoparticles and Their Use as Electrochromic Films. Inorg. Chem..

[4] Tong Z., Yang H., Na L., Qu H., Zhang X., Zhao J., Li Y. (2015). Versatile displays based on a 3-dimensionally ordered microporous vanadium oxide film for advanced electrochromic devices. J. Mater. Chem. C.

[5] Tang Y., Rui X., Zhang Y., Lim T.M., Dong Z., Hng H.H., Chen X., Yan Q., Chen Z. (2013). Vanadium pentoxide cathode materials for high-performance lithium-ion batteries enabled by a hierarchical nanoflower structure via an electrochemical process. J. Mater. Chem. A.

[6] Zhu J., Cao L., Wu Y., Gong Y., Liu Z., Hoster H.E., Zhang Y., Zhang S., Yang S., Yan Q. (2013). Building 3D structures of vanadium pentoxide nanosheets and application as electrodes in supercapacitors supercapacitors. Nano Lett..

[7] Wang Y., Cao G. (2006). Synthesis and Enhanced Intercalation Properties of nanostructured Vanadium Oxides. Chem. Mater..

[8] Marley P.M., Horrocks G.A., Pelcher K.E., Banerjee S. (2015). Transformers: The changing phases of lowdimensional vanadium oxide bronzes. Chem. Commun..

[9] D’Elia A., Cepek C., de Simone M., Macis S., Belec B., Fanetti M., Piseri P., Marcelli A., Coreno M. (2020). Interplay among work function, electronic structure and stoichiometry in nanostructured VO_x_ films. Phys. Chem. Chem. Phys..

[10] Mattelaer F., Geryl K., Rampelberg G., Dobbelaere T., Dendooven J., Detavernier C. (2016). Atomic layer deposition of vanadium oxides for thin-film lithium-ion battery applications. RSC Adv..

[11] Braithwaite J.S., Catlow C.R.A., Gale J.D., Harding J.H. (1999). Lithium intercalation into vanadium pentoxide: A theoretical study. Chem. Mater..

[12] Wu C., Xie Y. (2010). Promising vanadium oxide and hydroxide nanostructures: From energy storage to energy saving. Energy Environ. Sci..

[13] Wang Y., Cao G. (2008). Developments in Nanostructured Cathode Materials for High-Performance Lithium-Ion Batteries. Adv. Mater..

[14] Li H., He P., Wang Y., Hosono E., Zhou H. (2011). High-surface vanadium oxides with large capacities for lithium-ion batteries: From hydrated aerogel to nanocrystalline VO_2_(B), V_6_O_13_ and V_2_O_5_. J. Mater. Chem..

[15] Jiang J., Li Y., Liu J., Huang X., Yuan C., Lou X.W. (2012). Recent Advances in Metal Oxide-based Electrode Architecture Design for Electrochemical Energy Storage. Adv. Mater..

[16] Li L., Liu P., Zhu K., Wang J., Liu J., Qiu J. (2015). A general and simple method to synthesize well-crystallized nanostructured vanadium oxides for high performance Li-ion batteries. J. Mater. Chem. A.

[17] Kang W., Yan C., Wang X., Foo C.Y., Tan A.W.M., Chee K.J.Z., Lee P.S. (2014). Green synthesis of nanobelt-membrane hybrid structured vanadium oxide with high electrochromic contrast. J. Mater. Chem. C.

[18] Huang S.-Z., Cai Y., Jin J., Li Y., Zheng X.-F., Wang H.-E., Wu M., Chen L.-H., Su B.-L. (2014). Annealed vanadium oxide nanowires and nanotubes as high performance cathode materials for lithium ion batteries. J. Mater. Chem. A.

[19] Pang H., Dong Y., Ting S.L., Lu J., Li C.M., Kima D.-H., Chen P. (2013). 2D single- or double-layered vanadium oxide nanosheet assembled 3D microflowers: Controlled synthesis, growth mechanism, and applications. Nanoscale.

[20] Liu Y., Clark M., Zhang Q., Yu D., Liu D., Liu J., Cao G. (2011). V_2_O_5_ Nano-Electrodes with High Power and Energy Densities for Thin Film Li-Ion Batteries. Adv. Energy Mater..

[21] Uchaker E., Zheng Y.Z., Li S., Candelaria S.L., Hu S., Cao G.Z. (2014). Better than crystalline: Amorphous vanadium oxide for sodium-ion batteries. J. Mater. Chem. A.

[22] Armstrong E., McNulty D., Geaney H., O’Dwyer C. (2015). Electrodeposited Structurally Stable V_2_O_5_ Inverse Opal Networks as High Performance Thin Film Lithium Batteries. ACS Appl. Mater. Interfaces.

[23] Armstrong E., Osiak M., Geaney H., Glynn C., O’Dwyer C. (2014). 2D and 3D vanadium oxide inverse opals and hollow sphere arrays. CrystEngComm.

[24] Rolison D.R., Long J.W., Lytle J.C., Fischer A.E., Rhodes C.P., McEvoy T.M., Bourg M.E., Lubers A.M. (2009). Multifunctional 3D nanoarchitectures for energy storage and conversion. Chem. Soc. Rev..

[25] Caes S., Arrebola J.C., Krins N., Eloy P., Gaigneaux E.M., Henrist C., Cloots R., Vertruyen B. (2014). Mesoporous lithium vanadium oxide as a thin film electrode for lithium-ion batteries: Comparison between direct synthesis of LiV_2_O_5_ and electrochemical lithium intercalation in V_2_O_5_. J. Mater. Chem. A.

[26] Stein A., Wilson B.E., Rudisill S.G. (2013). Design and functionality of colloidal-crystal-templated materials—Chemical applications of inverse opals. Chem. Soc. Rev..

[27] Li H., Theriault J., Rousselle B., Subramanian B., Robichaud J., Djaoued Y. (2014). Facile fabrication of crack-free large-area 2D WO_3_ inverse opal films by a ‘dynamic hard-template’ strategy on ITO substrates. Chem. Commun..

[28] Li H., Vienneau G., Jones M., Subramanian B., Robichaud J., Djaoued Y. (2014). Crack-free 2D-inverse opal anatase TiO_2_ films on rigid and flexible transparent conducting substrates: Low temperature large area fabrication and electrochromic properties. J. Mater. Chem. C.

[29] Li H., Djaoued H., Robichaud J., Djaoued Y. (2020). A pleasant blue-green colored 2D Vanadium dioxide inverse opal monolayer: Large area fabrication and its thermochromic application. J. Mater. Chem. C.

[30] Li H., Wu H., Xiao J., Su Y., Robichaud J., Bruning R., Djaoued Y. (2016). A hierarchically porous anatase TiO_2_ coated-WO_3_ 2D IO bilayer film and its photochromic properties. Chem. Commun..

[31] Li H., Robichaud J., Djaoued Y. (2021). A simple way to fabricate pure anatase 2D TiO_2_ IO monolayer: Structure, color control and its application in electrochromism. RSC Adv..

[32] Baddour-Hadjean R., Smirnov M.B., Smirnov K.S., Kazimirov V.Y., Gallardo-Amores J.M., Amador M.E.U., Arroyo-de Dompablo J.P. (2012). Pereira-Ramos, Lattice Dynamics of β-V_2_O_5_: Raman Spectroscopic Insight into the Atomistic Structure of a High-Pressure Vanadium Pentoxide Polymorph. Inorg. Chem..

[33] Urena-Begara F., Crunteanub A., Raskin J.-P. (2017). Raman and XPS characterization of vanadium oxide thin films with temperature. Appl. Surf. Sci..

[34] Sahana M.B., Sudakar C., Thapa C., Naik V.M., Auner G.W., Naik R., Padmanabhan K.R. (2009). The effect of titanium on the lithium intercalation capacity of V_2_O_5_ thin films. Thin Solid Film.

[35] Wang Y., Takahashi K., Lee K., Cao G.Z. (2006). Nanostructured Vanadium Oxide Electrodes for Enhanced Lithium-Ion Intercalation. Adv. Funct. Mater..

[36] Chu S., Zhou L., Wang Z. (1991). A study of thermal decomposition of ammonium metavanadate. Eng. Chem. Metall..

[37] Goodenough J.B. (1970). Interpretation of M_x_V_2_O_5_-β and M_x_V_2__−*y*_T*_y_*O_5_-β phases. J. Solid State Chem..

[38] Wen T., Lu Z., Xu Z. (1994). Phase relationship and electrical conductivity of low valent vanadium-strontium oxide system. J. Inorg. Mater..

[39] Lu X.L. (2011). Photovoltaic Effect and Application of Vanadium Oxide. Master’s Thesis.

[40] Zhao X.K. (2007). The factors affecting absorption of IR spectrum. Inn. Mong. Petrochem. Ind..

[41] Talledo A., Granqvist C.G. (1995). Electrochromic vanadium–pentoxide–based films: Structural, electrochemical, and optical properties. J. Appl. Phys..

