# Supplementary material for: Two-Dimensional V2O5 Inverse Opal: Fabrication and Electrochromic Application
Source: Materials (Basel). 2022 Apr 15;15(8):2904. doi: 10.3390/ma15082904 (PMC9032571; doi:10.3390/ma15082904)
Supplement: Supplementary file 1 [file materials-15-02904-s001.zip › materials-1646175-supplementary.pdf]

2D V_2O_5 inverse opal：Fabrication and Electrochromic application

Hua Li[a, b], *Zijuan Tang*[a], *Yuwei Liu*[a], *Jacques Robichaud*[b],
Jian Liang[c], *Weihui Jiang*[c], *Yahia Djaoued*[b,*]

a, Department of Materials Chemistry, School of Materials Science and Engineering, Jingdezhen Ceramic University, Jingdezhen, Jiangxi, 333403, PR China, Tel: +86 798 8499678, E-mail: 201002@jci.edu.cn;

b, Laboratoire de Recherche en Matériaux et Micro-spectroscopies Raman et FTIR, Université de Moncton-Campus de Shippagan, Shippagan, NB, E8S1P6, Canada. Fax: +1 506 336 3434; Tel: +1 506 336 3412; E-mail: Yahia.djaoued@umoncton.ca;

c, National Engineering Research Centre for Domestic & Building Ceramics, Jingdezhen Ceramic Institute, Jingdezhen, Jiangxi, 333001, PR China, Tel: +86 798 8499328, E-mail: whj@jci.edu.cn;

Figures

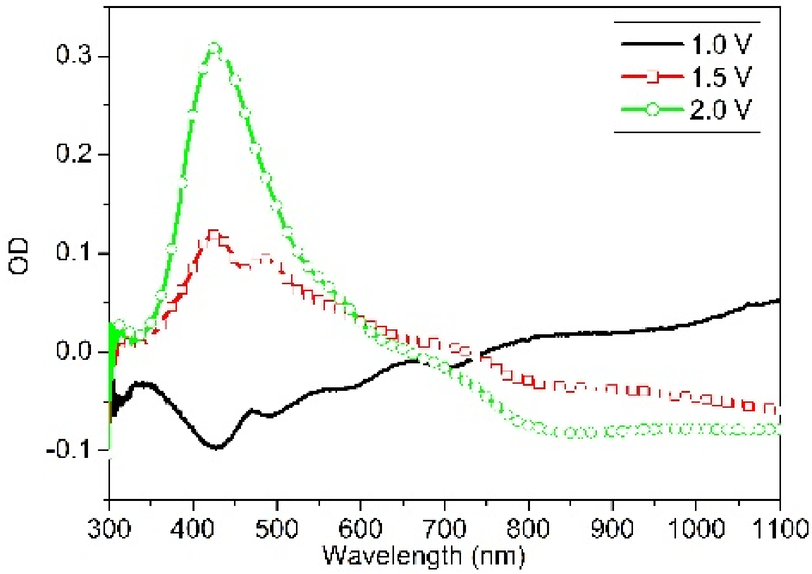

Figure S1 Optical density of V_2O_5 IO film under different applied voltages.
